# New Risk Factors for Transient Tachypnea of the Newborn and Childhood Asthma: A Study of Clinical Data and a Survey of Parents

**DOI:** 10.7759/cureus.6388

**Published:** 2019-12-15

**Authors:** Zuhal Gundogdu

**Affiliations:** 1 Pediatrics, Kocaeli University, Kocaeli, TUR

**Keywords:** transient tachypnea of newborn (ttn), childhood asthma, risk factors, elective cesarean, ttn therapy, allergic diseases

## Abstract

Objectives: It is established that transient tachypnea of the newborn (TTN) is associated with an increased risk of early childhood asthma. However, the question remains whether both asthma and TTN have common risk factors as well as the same underlying etiology. This study aims to determine possible risk factors for TTN as well as early childhood asthma.

Methods: This study was carried out in two phases. While the first phase included medical records of 1318 newborns, the second phase consisted of a phone survey.

Results: Elective cesarean section (ECS), maternal asthma, gestational age (GA), babies with large and small birth weight, number of pregnancies, and number of children were found to be significantly associated with TTN. ECS, maternal asthma, length of the hospital stay due to TTN, and O_2_ treatment were statistically significant for asthma. TTN was found to be associated with a subsequent diagnosis of childhood asthma after adjusting for ECS and maternal asthma.

Conclusions: Both ECS and maternal asthma are the common risk factors for the development of both TTN and childhood asthma as previously reported. In order to uncover this association, when ECS is taken out, it is seen that the association between TTN and asthma is stronger. Furthermore, O_2_ treatment and duration of hospital stay due to TTN were also found to be associated with childhood asthma. Association of maternal allergic rhinitis and eczema with TTN was investigated and there was no relationship between maternal allergic rhinitis or maternal eczema and the subsequent diagnosis of TTN.

## Introduction

Postnatal respiratory complications among newborns are common. One of the most commonly reported causes of neonatal respiratory distress is transient tachypnea of the newborn (TTN), with an estimated incidence of 1%-2% of all newborns [1].

Transient tachypnea of the newborn at birth is a condition in which fluid remaining in the lungs results in respiratory distress [2]. It is characterized by tachypnea shortly after birth, which clears within two to five days and shows itself by a typical clinical and radiographical presentation [3]. Once TTN resolves itself, there is usually no further increased risk of respiratory disease or other long-term sequelae [4]. Several previous studies showed that TTN is associated with an increased risk of childhood asthma [5-8].

Epidemiological studies showed that there is a relationship between TTN and the development of asthma in children [5-8]. This means that TTN alone is not only a condition itself but also a risk factor for future asthma in later childhood. Despite this, pinpointing a relationship between them is rather complicated due to etiology or risk factors. TTN and childhood asthma have been reported to be associated with cesarean section (CS) delivery [3, 5-9] and also pre-existing maternal asthma [3, 10-11]. Children with asthma are more likely to be delivered by CS [11] and infants born by elective CS (ECS) are at greater risk of developing respiratory problems, including TTN, compared with infants born vaginally [3-12].

This study examines the common etiology and risk factors for both TTN and early childhood asthma. It also attempts to determine whether TTN is the first manifestation of asthma in early childhood.

## Materials and methods

Study population

Data were collected from the medical records of children born at Akademi Hospital in Kocaeli, Turkey from January 2007 to April 2012 and admitted to a special neonatal intensive care unit. Demographic and clinical information, about mothers and children included date of birth, gestational age (GA) at birth, birth weight, gender, number of pregnancies and number of children in the family, mothers’ allergic diseases (asthma, allergic rhinitis, and eczema), contact details of parents, and mode of delivery (normal spontaneous vaginal delivery, NSVD or CS). If the delivery was by CS, further information such as whether it was an ECS or emergency was obtained.

**Figure 1:**
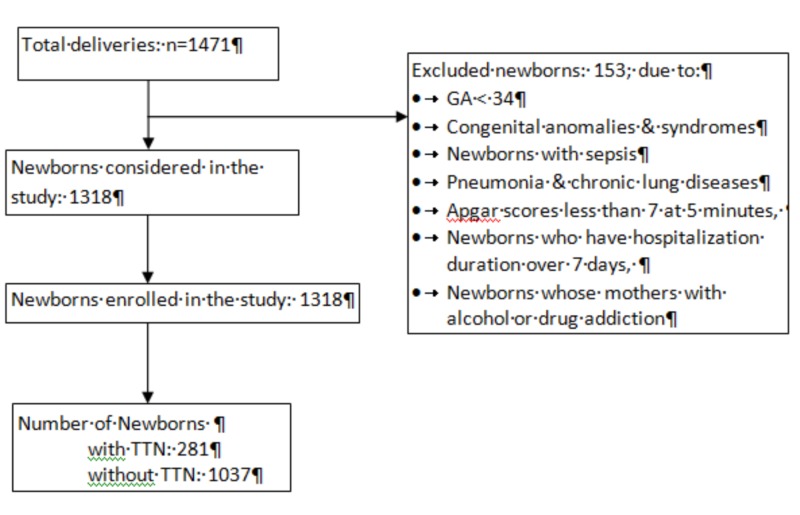
Flow diagram of the study.

There were 1471 live births between 2007 and 2012. One hundred and fifty-three newborns with GA less than 34 weeks, congenital anomalies and syndromes, sepsis, pneumonia, chronic lung diseases, Apgar scores less than 7 at 5 min, newborns who have hospital stay duration over seven days, and whose mothers with alcohol or drug addiction were excluded from the study (Figure 1). The first part of the study thus included 1318 newborns. 

Gestational age at birth was defined as complete weeks of gestation based on the estimated date of delivery in each woman’s clinical record. The GA of 34-42 weeks at birth was selected. Children with GA <34 weeks were excluded because newborns less than this GA have a higher risk of respiratory and other health complications. GAs were determined based on maternal last menstrual period or early prenatal ultrasound examination findings [1].

Birth weight (in kg) was measured after delivery using the same equipment for each baby. The birth weight of all newborns was separated into three groups: large for gestational age (LGA), small for gestational age (SGA), and appropriate for gestational age (AGA) according to GA scale. Birth weight of all newborns was separated into three groups; LGA if the birth weight is greater than 90th percentile, SGA if the birth weight is smaller than 10th percentile, and AGA for between these two according to GA scale [13].

Cesarean section was defined as elective when surgery was performed before the onset of labor, whereas all the other cases were defined as nonelective CS due to medical reasons. The number of pregnancies and the number of children were classified into two groups: three or above and under three. Consent was obtained in order to access medical records for the first part of this study. This study was approved by the local hospital committee as, at the time of this study, no ethical approval was needed for retrospective studies.

A comparative study has been carried out between those who had TTN and those without TTN. This study also looked into the type of respiratory support (i.e. O_2_ or mechanical ventilation) and the length of the hospital stay within the TTN group.

**Figure 2:**
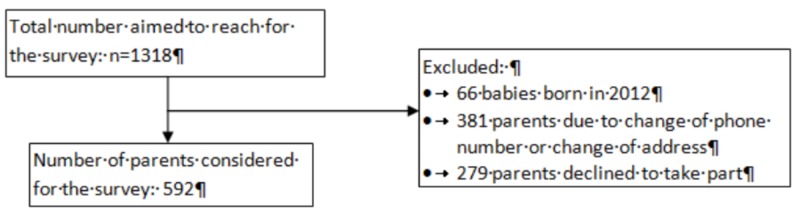
Survey flow diagram.

The second part of this study was carried out between June and August 2012 and involved a phone survey of the parents. Among 1318 births, 66 babies born in 2012 were excluded from the second part of the study as they would be around six months old at the time of the phone survey and too young for wheezing infant diagnosis. The phone survey included phase two core questionnaires from The International Study of Asthma and Allergies in Childhood (ISAAC) [14]. Out of 1252 parental records, 592 parents were finally included in the second part of the study. Figure 2 shows the flow diagram of the survey. 

The purpose of the study was clearly explained to them, and they were reassured that the information they supply would remain confidential. Furthermore, parents were also told that taking part in the survey was purely voluntary. Survey questions were read out word-by-word from the survey document to each parent and their answers were recorded.

Parents were asked whether their children have been diagnosed with asthma or not. While some parents were certain that their children had asthma since their children were diagnosed with it by a pediatrician or pediatric asthma and allergy specialist, some were uncertain about whether their children have asthma. It is for this reason their children were further evaluated through questions from ISAAC phase two core questionnaires.

Statistical analysis

SPSS (Statistical Package for Social Science), version 20, was used for statistical analysis. Potentially confounding variables such as GA, birth weight, number of pregnancies, number of children, maternal allergic diseases, mode of delivery, gender, O_2_ treatment, mechanical ventilation, and the length of the hospital stay in relation with either TTN or childhood asthma were evaluated using either chi-square or Mann-Whitney test.

Logistic regression was used to assess the effect of the dependent variable; TTN, ECS, and number of children three or more, and childhood asthma. In order to explore the presence of suspected causal association of TTN and childhood asthma, multivariate analysis of subgroups was also carried and an adjustment was made with factors that were related to asthma.

## Results

The first part of the study included 1318 newborns with all the relevant maternal and neonatal data and out of these 281 (21.3%) children had developed TTN at birth.

Demographic characteristics such as GA, date of birth, birth weight, gender, mode of delivery, whether elective CS or not, number of pregnancies and children, any maternal allergic diseases (asthma, allergic rhinitis, eczema, etc.), newborns with or without TTN are summarized and compared between the group with TTN and without TTN as shown in Table 1.

**Table 1:** Demographics of newborns with and without TTN. TTN, tachypnea of the newborn; SD, standard deviation; LGA, large for gestational age; AGA, appropriate for gestational age; SGA, small for gestational age; NSVD, normal spontaneous vaginal delivery; ECS, elective cesarean section.

Demographics total	Newborn without TTN	Newborn with TTN	p-value
Number	1037	281	
Gestational age, weeks (mean ± SD)	38.66±1.14	37.71±1.40	<0.001
Birth weight, kg (mean ± SD)	3.23±0.44	3.11±0.57	0.001
LGA (%)	12.4	17.4	
AGA (%)	83.7	73.0	
SGA (%)	2.1	3.9	
Male sex (%)	52	56.6	0.179
NSVD (%)	17.7	7.5	<0.001
ECS (%)	16.3	67.3	<0.001
Maternal asthma (%)	1.2	3.6	0.006
Maternal allergic rhinitis (%)	2.5	1.4	0.271
Maternal eczema (%)	1.0	0.7	0.683
Number of pregnancies (%)	4.9 (≥ 3)	11.5 (≥ 3)	<0.001
Number of children (%)	3.6(≥ 3)	10.8 (≥ 3)	<0.001

Transient tachypnea of the newborn changed inversely with GA and decreased with advancing GA from 37 completed weeks and onwards. Table 1 also shows that the overall frequency of ECS was 67.3% among newborns with TTN, compared to 16.3% those without TTN (p<0.001). Some variables (number of pregnancies, number of children greater than three, NSVD, ECS, and maternal asthma) were also found to be significantly associated with the diagnosis of TTN. However, maternal allergic rhinitis and eczema were not risk factors for TTN.

The second phase of the study was performed among the parents of those 592 children. The demographic characteristics of this study group are presented in Table 2 and comparisons were made between children with asthma and without asthma. The newborns with TTN, born with ECS and maternal asthma were at increased risk of developing asthma in later childhood (Table 2). In addition, O_2_ treatment and duration of hospital stay due to TTN were also found to be associated with childhood asthma (p<0.001). However, birth weight, GA, maternal eczema, allergic rhinitis, number of pregnancies and children three and above were not associated with childhood asthma.

**Table 2:** Demographics of children with asthma and without asthma from the phone survey data. SD, standard deviation; LGA, large for gestational age; AGA, appropriate for gestational age; SGA, small for gestational age; ECS, elective cesarean section; TTN, transient tachypnea of the newborn.

Demographics	Children without asthma	Children with asthma	p-value
Number	528	64	
Gestational age (mean ± SD)	38. 41 ± 1. 30	38.34 ± 1.29	0.823
Birth weight (mean ± SD)	3. 22 ± 0. 49	3. 16 ± 0.49	0.906
LGA (%)	14.2	15.6	
AGA (%)	79.7	76.6	
SGA (%)	3.4	3.2	
Male sex (%)	51.3	60.9	0.146
ECS (%)	27.1	43.8	0.004
Maternal asthma (%)	2.8	9.4	0.008
Maternal allergic rhinitis (%)	3.2	6.3	0.216
Maternal eczema (%)	1.3	0	0.354
Number of pregnancies (%)	7.6 (>=3)	9.4 ( >=3)	0.612
Number of children (%)	8.8 (>=3)	6.3 ( >=3)	0.501
TTN (%)	21.6	48.4	<0.001
O_2_ treatment without mechanical ventilation (%)	21.0	46.9	<0.001
Mechanical ventilation (%)	0.6	1.6	0.360
Duration of hospital stay (%)	7.2 (>3day<7day)	14(>3day<7day)	<0.001

Multiple logistic regressions were used to investigate the causal relationship between TTN and childhood asthma. The analysis was adjusted for maternal asthma and ECS, which are known to be associated with the development of both childhood asthma and TTN. Table 3 shows the results of this association. TTN was found to be associated with a subsequent diagnosis of childhood asthma after adjusting for ECS and maternal asthma. The adjusted odds ratio was 0.35 (95% CI: 0.181-0.678. p=0.002). The effect of maternal asthma on childhood asthma was found to be borderline statistical significance (p=0.05), while elective CS was not statistically significant (p=0.38).

**Table 3:** Multiple logistic regression analysis of the association of TTN and childhood asthma. OR, odds ratio; CI, confidence interval; CS, cesarean section; TTN, transient tachypnea of the newborn.

	Adjusted OR	95% CI	p-value
Elective CS	1.347	0.695-2.609	0.377
Maternal asthma	3.128	1.011-9.675	0.048
TTN	0.350	0.181-0.678	0.002

## Discussion

Main findings

Demographic characteristics of newborns at birth were examined for the development of TTN, including birth weight, GA, maternal asthma, CS, ECS, number of pregnancies, and number of children in the family.

Cesarean section has been well described as being a risk factor for TTN because of the absence of a surge in catecholamines normally released in a vaginal delivery, which may play a role in the adequate and timely clearance of lung fluid similar to the trigger effect of labor [4,12,15-18]. A similar relationship between ECS and TTN was also observed in a population of 1318 children in this study. In line with results from previous studies, TTN incidence is inversely related to the GA [12,16-18].

In this study, newborns were grouped as AGA, SGA, and LGA according to their birth weight and GA by using reference percentile values. LGA and SGA appear to be associated with increased TTN. Additionally, while male sex and LGA have also been associated with increased TTN in literature [4,15], there was no such relationship with TTN in this study. 

Several studies have found that maternal asthma is also a risk factor for TTN. Demissie et al. used a historical cohort analysis and found that maternal asthma is a risk factor for TTN as also reported [11].

Association of maternal allergic rhinitis and eczema with TTN was for the first time investigated in this study and it was found that there was no relationship between maternal allergic rhinitis or maternal eczema and subsequent diagnosis of TTN in our study.

Transient tachypnea of the newborn therapy is also identified as a risk factor in the development of early childhood asthma. In contrast to other studies, this study shows that hospital duration and respiratory support treatment, especially O_2_ treatment without mechanical ventilation have an effect on a child developing asthma.

Liem et al. reported that those infants diagnosed with TTN being treated with intravenous antibiotics for at least 48 h before blood cultures as negative may have had modified gastrointestinal flora, which then would no longer provide flora protective influence on the development of allergy and asthma [4]. In this study, newborns with TTN were not given any antibiotic treatment. Hospitalization duration and O_2_ treatment might have changed the flora of the newborns which might have caused childhood asthma. However, we could not explain that mechanical ventilation treatment does not have any effect on having childhood asthma in our results.

This study shows that the number of children three and above as well as the number of pregnancies three and above led to an increased risk of TTN. However, these are not significant in relation to childhood asthma. No previously reported studies have investigated the association of the number of children and the number of pregnancies for TTN. Pregnancy might be an indicator of the socioeconomic level. It is well known that demographic and environmental characteristics such as race and urban domicile are associated with asthma [3-4].

Previous studies had demonstrated an increased risk of asthma among children delivered by CS [5]. In our study, TTN and childhood asthma are both seen in infants delivered by ECS. In that case, one needs to discuss whether ECS is a common factor for both TTN and childhood asthma or the two of them are independent. In order to uncover this association, it is seen that the association between TTN and asthma is stronger when ECS is taken out.

Strengths and limitations of this study

Being able to follow up information on children since their birth with the added strength of a survey evaluating their later childhood respiratory problems and family details are the main strengths of this study. Patient records studied here had all the relevant maternal data throughout pregnancy as well as prenatal and postnatal information. However, although a fairly large population of clinical records was studied, a smaller number of parents during the phone survey were reached. 

Interpretation of findings in relation to previously published work

It was found that TTN is a risk factor for a future wheezing syndrome and it may not be as transient as previously thought as several studies have shown an association between TTN and the development of asthma [4-5,19].

This study is different, as a survey of all of the parents whether their children had TTN or whether their children have childhood asthma or not has been carried out. Furthermore, the risk factors have been determined for childhood asthma and TTN. This study is the first study that maternal allergic rhinitis and eczema are investigated as possible risk factors in relation to TTN. It was shown that O_2_ treatment and hospital duration are significant factors for childhood asthma as these might be risk factors and should further be investigated.

This study showed that, after controlling for possible confounding factors by ECS delivery, TTN, which was markedly stronger, was found to be independently associated with the subsequent diagnosis of childhood asthma similar to a study reported in Ref. [3].

Further investigation of other potential risk factors such as maternal asthma, GA, number of pregnancies, and number of children showed that the association of TTN and the subsequent diagnosis of asthma were especially strong among infants whose mothers did not have ECS as well as infants whose mothers did not have asthma.

Our results also reconfirm that TTN is associated with childhood asthma. TTN may be an important indicator of impaired lung function in early childhood and this abnormality of lung function may be what makes the same infant who is susceptible to TTN also at risk of developing asthma [3].

Birnkrant et al. state that complex diseases such as asthma will result from the dynamic interaction of an abnormal respiratory system with many and varied environmental exposures such as allergens, irritants, and infections [3]. Obesity is also a risk factor for reduced airflow or lung function [20].

Further studies should address the more complex relationship between TTN and asthma managed in the community. The follow-up period in our study was limited to childhood like many other studies. Recent studies have reported a greater than threefold risk of asthma in adulthood due to cesarean delivery. The possible role of neonatal respiratory morbidity in the etiology of adult asthma remains to be determined [5].

## Conclusions

This two phase study found that TTN is associated with respiratory problems and may be an indication of asthma in later life. Furthermore, maternal allergic rhinitis and eczema, number of pregnancies, and number of children three and above were investigated in relation to the development of TTN and childhood asthma. TTN and asthma are both seen in infants delivered by ECS and another common risk factor is maternal asthma. One needs to discuss whether ECS is a common factor for both TTN and later childhood asthma or they are two independent diseases. In order to uncover this association, when ECS is taken out, it is seen that the association between TTN and asthma is stronger. In addition, O_2_ treatment and duration of hospital stay due to TTN were also found to be associated with childhood asthma. Association of maternal allergic rhinitis and eczema with TTN was for the first time investigated and there was no relationship between maternal allergic rhinitis or maternal eczema and the subsequent diagnosis of TTN. TTN therapy is also identified as a risk factor in the development of early childhood asthma. In contrast to other studies, this study shows that hospital duration and respiratory support treatment, especially O_2_ treatment without mechanical ventilation have an effect on the child developing childhood asthma and should further be investigated.
